# 2655. Phase 1 Safety and Immunogenicity Results of Two Investigational mRNA Vaccines, mRNA-1345, a Respiratory Syncytial Virus Vaccine, and mRNA-1653, a Human Metapneumovirus and Parainfluenza Virus Type 3 Combination Vaccine in Seropositive Young Children

**DOI:** 10.1093/ofid/ofad500.2266

**Published:** 2023-11-27

**Authors:** Matthew D Snape, Sabine Schnyder Ghamloush, Grace L Chen, Rakesh Dhar, Runa Mithani, Vinicius Righi, Louie Morsy, Archana Kapoor, Bethany Girard, Laila El Asmar, Christine A Shaw

**Affiliations:** Moderna Biotech UK, Inc, Didcot, England, United Kingdom; Moderna, Inc., Cambridge, Massachusetts; Moderna, Inc., Cambridge, Massachusetts; Moderna, Inc., Cambridge, Massachusetts; Moderna, Inc., Cambridge, Massachusetts; Moderna, Inc., Cambridge, Massachusetts; Moderna, Inc., Cambridge, Massachusetts; Moderna, Inc., Cambridge, Massachusetts; Moderna, Inc., Cambridge, Massachusetts; Moderna, Inc., Cambridge, Massachusetts; Moderna, Inc., Cambridge, Massachusetts

## Abstract

**Background:**

Respiratory syncytial virus (RSV), human metapneumovirus (hMPV), and parainfluenza virus type 3 (PIV3) are common respiratory illnesses in children. Two investigational vaccines, mRNA-1345, encoding the RSV prefusion stabilized F (preF) glycoprotein, and mRNA-1653, encoding the hMPV and PIV3 F glycoproteins, are in clinical trials.

**Methods:**

Two phase 1, randomized, observer-blind, placebo-controlled trials in children aged 12-59 months assessed safety and immunogenicity of mRNA-1345 (NCT04528719) and mRNA-1653 (NCT04144348). In the mRNA-1345 trial, RSV-seropositive children (N=46) were randomized to receive 3 doses of mRNA-1345 (15 µg or 30 µg) or placebo 2 months apart. In the mRNA-1653 trial, hMPV- and PIV3-seropositive children (N=27) were randomized to receive 2 doses of mRNA-1653 (10 µg or 30 µg) or placebo 2 months apart. Interim data through Month (M) 5 for mRNA-1345 and M3 for mRNA-1653 are reported.

**Results:**

mRNA-1345 and mRNA-1653 were well-tolerated. The most frequently reported solicited local adverse reaction (AR) was tenderness at injection site (mRNA-1345, 35.7%-71.4%; placebo, 26.7%-42.9% and mRNA-1653, 44.4%-60.0%; placebo, 12.5%-30.0%); solicited systemic ARs (mRNA-1345, 12.5%-53.3%; placebo, 33.3%-50.0% and mRNA-1653, 33.3%-55.6%; placebo, 12.5%-60.0%) were mostly grade 1/2. One mRNA-1345 injection boosted RSV neutralizing antibody (nAb) titers (geometric mean fold rise [GMFR] over baseline: RSV-A=18.9-34.9; RSV-B=7.2-14.3) and RSV preF and postF binding antibody (bAb) concentrations (GMFR: preF=13.9-26.5; postF=9.3-16.0); additional injections did not further elevate antibody levels (**Fig 1A and 2A**). One mRNA-1653 injection boosted hMPV and PIV3 nAb titers (GMFR over baseline: hMPV-A=2.9-6.1; hMPV-B=6.2-13.2; PIV3=2.8-3.0) and preF and postF bAb concentrations (GMFR: hMPV preF=5.3-6.1; postF=4.6-6.5 and PIV3 preF=13.9-14.2; postF=11.0-12.1); a second injection did not further increase antibody levels (**Fig 1B,C and 2B,C**). In both trials, bAb responses were generally preF biased.

**Figure:**
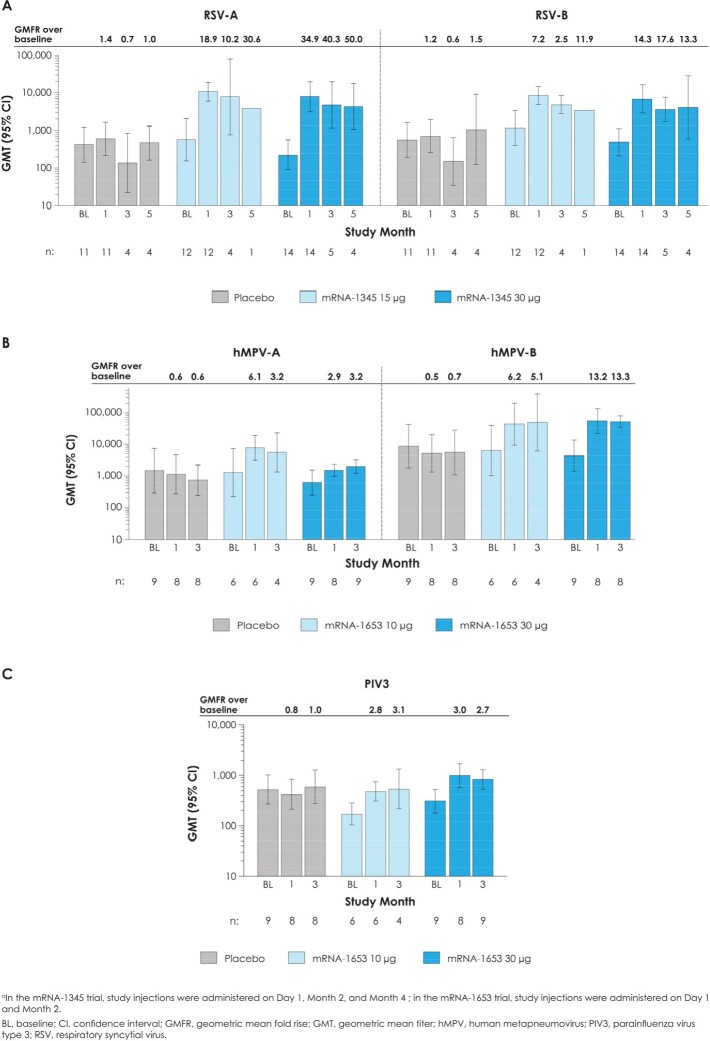

**Figure:**
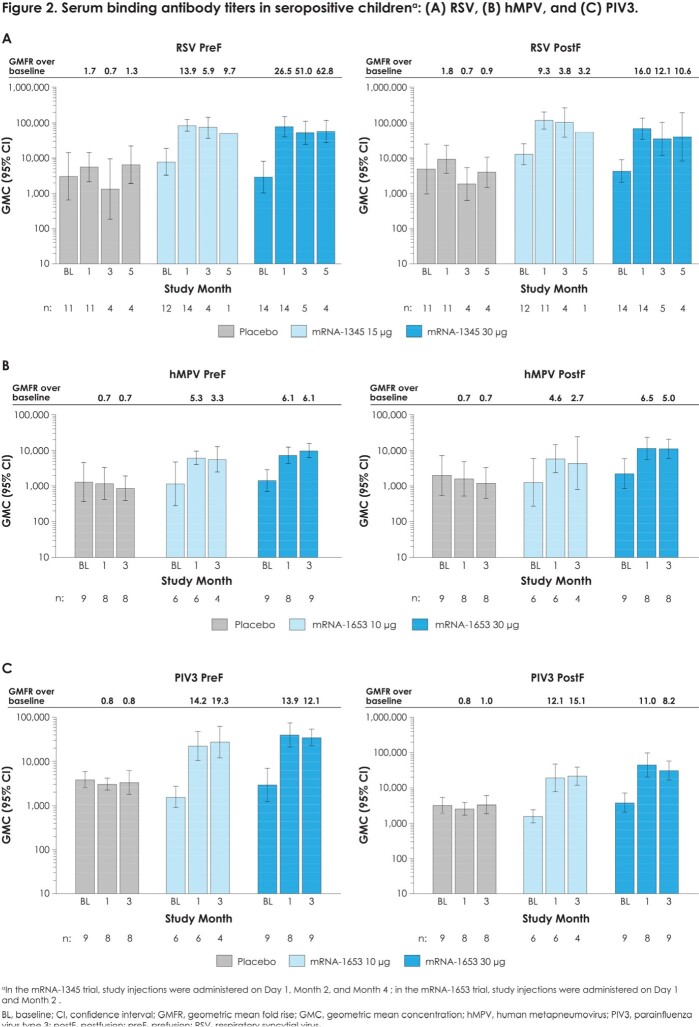

**Conclusion:**

In seropositive children aged 12-59 months, mRNA-1345 and mRNA-1653 were well-tolerated and boosted RSV and hMPV plus PIV3 antibodies, respectively, supporting their continued development and that of a combination RSV and hMPV vaccine.

**Disclosures:**

**Matthew D. Snape, MBBS, MD, FRCPCH, FPM, FMedSci**, Moderna Biotech UK, Inc.,: Employee|Moderna Biotech UK, Inc.,: Stocks/Bonds **Sabine Schnyder Ghamloush, MD**, Moderna, Inc.: Employee|Moderna, Inc.: Stocks/Bonds **Grace L. Chen, MD, MPH**, Moderna, Inc.: Employee|Moderna, Inc.: Stocks/Bonds **Rakesh Dhar, MD**, Moderna, Inc.: Employee|Moderna, Inc.: Stocks/Bonds **Runa Mithani, PharmD**, Moderna, Inc: Employee|Moderna, Inc: Stocks/Bonds **Vinicius Righi, PharmD, MBA**, Moderna, Inc.: Employee|Moderna, Inc.: Stocks/Bonds **Louie Morsy, BS**, Moderna, Inc.: Employee|Moderna, Inc.: Stocks/Bonds **Archana Kapoor, PhD**, Moderna, Inc.: Employee|Moderna, Inc.: Stocks/Bonds **Bethany Girard, Ph.D.**, Moderna, Inc.: salary|Moderna, Inc.: Stocks/Bonds **Laila El Asmar, PhD**, Moderna, Inc.: Employee|Moderna, Inc.: Stocks/Bonds **Christine A. Shaw, PhD**, Moderna, Inc.: Employee|Moderna, Inc.: Stocks/Bonds

